# Machine Learning Insights Into Uric Acid Elevation With Thiazide Therapy Commencement and Intensification

**DOI:** 10.7759/cureus.51109

**Published:** 2023-12-26

**Authors:** Murat Özdede, Alper T Guven

**Affiliations:** 1 Internal Medicine, Hacettepe University Faculty of Medicine, Ankara, TUR; 2 Internal Medicine, Baskent University Faculty of Medicine, Ankara, TUR

**Keywords:** hydrochlorothiazide, indapamide, thiazides, machine learning, hyperuricemia, hypertension

## Abstract

Background

Elevated serum uric acid, associated with cardiovascular conditions such as atherosclerotic heart disease, hypertension, and heart failure, can be elevated by thiazide or thiazide-like drugs (THZ), essential in hypertension management. Identifying clinical determinants affecting THZ-related uric acid elevation is critical.

Methods

In this retrospective cross-sectional study, we explored the clinical determinants influencing uric acid elevation related to THZ, focusing on patients where THZ was initiated or the dose escalated. A cohort of 143 patients was analyzed, collecting baseline and control uric acid levels, alongside basic biochemical studies and clinical data. Feature selection was conducted utilizing criteria based on mean squared error increase and enhancement in node purity. Four machine learning algorithms - Random Forest, Neural Network, Support Vector Machine, and Gradient Boosting regressions - were applied to pinpoint clinical influencers.

Results

Significant features include uncontrolled diabetes, index estimated Glomerular Filtration Rate (eGFR) level, absence of insulin, action of indapamide, and absence of statin treatment, with absence of Sodium-glucose cotransporter 2 inhibitors (SGLT2i), low dose aspirin exposure, and older age also being noteworthy. Among the applied models, the Gradient Boosting regression model outperformed the others, exhibiting the lowest Mean Absolute Error (MAE), Mean Squared Error (MSE), Root Mean Squared Error (RMSE) values, and the highest R2 value (0.779). While Random Forest and Neural Network regression models were able to fit the data adequately, the Support Vector Machine demonstrated inferior metrics.

Conclusions

Machine learning algorithms are adept at accurately identifying the factors linked to uric acid fluctuations caused by THZ. This proficiency aids in customizing treatments more effectively, reducing the need to unnecessarily avoid THZ, and providing guidance on its use to prevent instances where uric acid levels could become problematic.

## Introduction

Uric acid, an end product of the metabolic breakdown of purine nucleotides, plays a crucial role in human health, with abnormal levels being associated with various medical conditions, including gout, kidney stones, metabolic syndrome, chronic kidney disease (CKD), diabetes mellitus (DM) and atherosclerotic heart disease [1]. High levels of uric acid in the serum are frequently associated with hypertension, and a positive correlation exists in patients who have metabolic syndrome. This association presents a causality dilemma and plays a role in elevating the risk of cardiovascular diseases [2, 3]. This is likely due to the shared underlying mechanisms that lead to hypertension and cardiovascular diseases, and their association with elevated uric acid levels [4, 5]. The relationship between uric acid and hypertension is complex, with proposed mechanisms highlighting the roles of inflammation, oxidative stress, and arterial stiffness leading to atherosclerosis [6-9].

Thiazide or thiazide-like diuretics (THZ) are highly regarded antihypertensive medications, considered 'old but gold.' Their prominent status stems not only from their efficacy in managing blood pressure and reducing cardiovascular morbidity and mortality, as supported by meta-analyses, but also from their associated low rates of nonadherence [10-13]. However, while they are frequently prescribed for hypertension and associated with favorable cardiac outcomes, the initiation or escalation of these medications can notably impact uric acid levels, which are linked with unfavorable cardiac events, thus presenting a clinical conundrum [14, 15].

Although the relationship between uric acid levels and THZ is well-established, there remains a significant lack of detailed understanding of the clinical factors that dictate uric acid variations following the initiation or intensification of THZ. A detailed evaluation of these influential elements is crucial in promoting the best clinical practices, individualized treatment strategies, and improved patient care, particularly in groups with hypertension. Motivated by this, this paper aims to evaluate the crucial factors affecting changes in uric acid levels related to THZ, utilizing sophisticated machine learning methods to examine contributing and alleviating factors and to furnish insights into predicting the magnitude of change.

## Materials and methods

Design, setting and the study population

This study employed a subset of patients derived from the dataset of our earlier research, acting as the foundation for this study [16]. This cohort comprises cases from four distinct clinics, both secondary and tertiary care centers, spanning from October 1st, 2020, to October 30th, 2021. The dataset had undergone rigorous cleaning and mild imputation processes as outlined by our prior study's exclusion criteria. Subsequently, this research focused on individuals for whom THZ drugs were either initiated or escalated and involved those with available index and control uric acid levels. Patients who were treated or initiated with losartan were excluded due to its uric acid-lowering properties, alongside those administered Xanthine Oxidase inhibitors and those with acute kidney injuries, to eliminate their influences on THZ-associated uric acid changes.

Our study included a post-hoc power analysis, executed using G*Power software version 3.1.9.4, to determine the study's power. The alpha level was established at 0.05, and the total sample size was determined following the exclusion of data from our dataset. The effect size was computed based on the average impact of codeterminants on uric acid levels.

Case definition and data acquisition

Cases were required to have undergone two visits: an index visit, where the intervention took place, and a control visit, occurring in four weeks, where blood studies were reassessed. The intervention or "the action" involved decisions related to the initiation or escalation of hydrochlorothiazide or indapamide. In the context of our study, which focuses on Turkey, hydrochlorothiazide is solely available in combination pills. Meanwhile, indapamide is available both as a standalone medication and in combination with perindopril. Chlortalidone, typically available only in combination with atenolol, was not present in our cohort. Therefore, in this study, the only thiazide-like diuretic was indapamide, and it was abbreviated along with hydrochlorothiazide as "THZ".

Each patient’s data included demographic information, comorbidities, current medications, laboratory measurements, and details about actions taken regarding antihypertensive classes. Specific conditions and diseases were defined based on established diagnoses and guidelines, and active cancer was identified based on recent diagnoses or treatments. Diabetes mellitus was defined as self-reported DM diagnoses or via anti-diabetic medication use. As a subgroup, uncontrolled DM was arbitrarily defined as patients with an A1c over 8%. Detailed definitions of specific diseases are available from our previous research [16].

Concerning renin-angiotensin-aldosterone system inhibitors (RAASi), β-blockers (βbl), or calcium channel blockers (CCb), a medication from these classes may have been newly initiated or might have already been in use. If in use, their administration could have been ceased, or their doses could have been adjusted - either increased, decreased, or maintained at the existing level. For the purpose of comparative analysis, the initiation of a drug from these classes was grouped with the same drug’s dose escalation and the cessation was grouped with dose reduction, due to their anticipated similar impacts on uric acid levels. In cases where a drug was not introduced to patients who were naïve to it, those instances were grouped with cases where the same drug dose remained unchanged.

For laboratory parameters, serum uric acid levels acquired at the index visit (pre-action) were labeled as 'index uric acid.' Those obtained at the control visit (post-action) were denoted as 'control uric acid.' The numeric target variable was represented as 'Δuric acid' and was calculated by subtracting index levels from control levels.

Descriptive statistics

Continuous variables are presented as mean ± standard deviation, as they are all normally distributed. The Student's t-test or ANOVA was used for analyzing differences between normally distributed continuous variables, with Levene’s test assessing the equality of variances for each variable calculated across groups. Categorical variables are represented as counts and percentages, analyzed using the chi-squared (χ2) or Fisher’s exact test as appropriate. The relationship between the increase in uric acid levels, baseline uric acid values, and clinical characteristics is determined using the Pearson's correlation test. All conventional statistical analyses were conducted utilizing corresponding functions and ‘psych’ package in R 4.2.2 programming language.

Feature selection

Before developing the regression models, a feature selection process was conducted to eliminate unnecessary, irrelevant, and redundant features. This study employed a methodical approach, selecting eight features based on their linear relationship with the target variable, assessed using correlation coefficients, and on model-based feature importance scores. The importance of each feature was evaluated using random forest regression, focusing on the increase in mean squared error and improvement in node purity, to enhance the understanding of each feature's relevance. This approach allowed the inclusion of impactful features, omitting the less significant ones without compromising predictive accuracy. The feature selection was carried out using the randomForest() package in R 4.2.2.

Machine learning algorithms

Four machine learning algorithms were employed to predict THZ-associated uric acid changes. Given that the numeric target variable converts the classification into a regression problem, we opted for Random Forest Regression (RFreg), Gradient Boosting Regression (GBreg), Support Vector Machine Regression (SVMreg), and Neural Network Regression (NNreg). For SVMreg, the Radial Basis Function (RBF) kernel was used. Both RFreg and GBreg were configured to create 200 decision trees, and NNreg was set with 250 neurons in hidden layers, utilizing a Rectified Linear Unit (ReLU) activation function and the Limited-memory Broyden-Fletcher-Goldfarb-Shanno (L-BFGS-B) optimization algorithm.

Machine learning algorithms were implemented using Orange 3.34.0 Software (University of Ljubljana, Slovenia), and each model’s performance was evaluated through the software’s Test and Score module using Mean Squared Error (MSE), Root Mean Squared Error (RMSE), Mean Absolute Error (MAE), and adjusted R2. The former two quantify the average magnitude of the residuals or prediction errors; the third assesses the fit of the model, and the latter represents the average absolute difference between the observed actual outcomes and the predictions made by the model.

## Results

Patient selection

**Figure 1:**
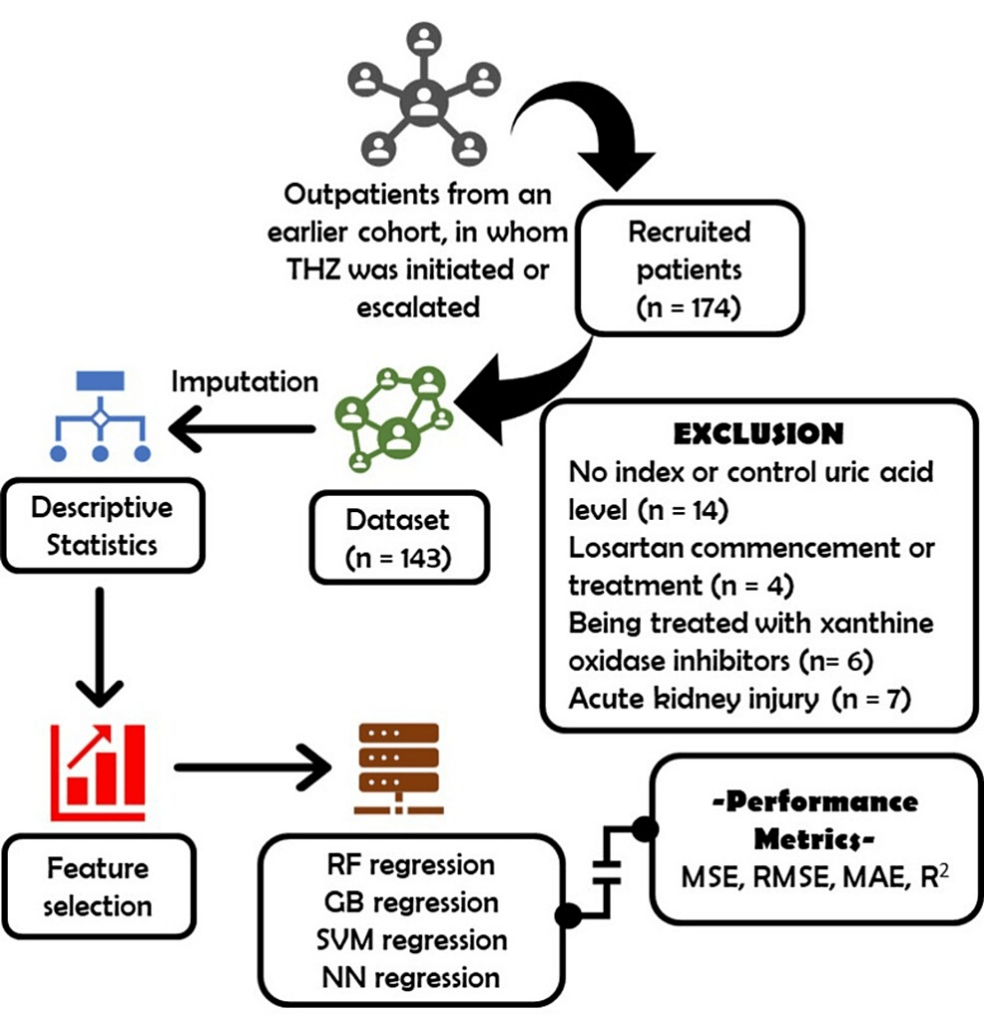
Flowchart of the study GB: Gradient Boosting, MAE: Mean Absolute Error, MSE: Mean Squared Error, NN: Neural Network, RMSE: Root Mean Squared Error, R2: adjusted R2, RF: Random Forest, SVM: Support Vector Machine, THZ: Thiazide diuretics

From the initial dataset, 174 patients were identified as having either initiated or escalated THZ. Among these, 14 lacked both index and control uric acid levels. Four had undergone treatment with or had started losartan treatment. Six had records of allopurinol, and seven experienced acute kidney injury, which was the principal area of investigation in our preceding study, leading to their exclusion (Figure 1). A total of 143 patients were found to be eligible and included in the study.

Clinical data and laboratory parameters

The cohort studied displayed a modest male dominance, constituting 65.7% (n = 94), and 52.4% were aged above 60, as shown in Table 1. Diabetes Mellitus (DM) was the prevalent comorbidity, appearing in 43.4% (n = 62) of cases, with metformin being the predominant medication, used by 35% (n = 50). Table 1 reveals that both initial and Δuric acid levels maintained uniformity across genders and age groups. Diabetes didn't appear to impact these levels; however, uncontrolled diabetes mellitus revealed a notably higher Δuric acid (1.17 ± 1.12; p=0.003). Patients with coronary artery disease and chronic kidney disease presented higher initial uric acid levels (5.8 ± 1.4; p=0.005 and 6.8 ± 1.12; p=0.001, respectively) but the Δuric acid levels were consistent with the rest of the cohort.

**Table 1:** Index uric acid and Δuric acid levels according to demographic and clinical characteristics THZ: Thiazide & Thiazide like Diuretics, RAASi: Renin-Angiotensin-Aldosterone System Inhibitors; NSAID: Non-steroidal anti-inflammatory drugs, SGLT2i: Sodium-Glucose Transporter-2 inhibitors, CCb: Calcium Channel Blockers which consist of dihydropyridines and nondihydropyridines, β-blockers: Beta-blockers

	Total	Index Uric Acid	Sig.	Δuric acid	Sig.
Total Cohort	133 (100%)	5.2 ± 1.3		0.6 ± 0.87	
Demographic Status					
Age > 60	75 (52.4%)	5.13 ± 1.3	0.54	0.61 ± 0.82	0.87
Female	49 (34.3%)	5.12 ± 1.4	0.3	0.52 ± 0.7	0.17
Comorbidities					
Diabetes Mellitus	62 (43.4%)	5.4 ± 1.4	0.07	0.64 ± 1.03	0.6
Uncontrolled Diabetes Mellitus	18 (12.6%)	5.1 ± 1.8	0.8	1.17 ± 1.12	0.003
Coronary Artery Disease	28 (19.6%)	5.8 ± 1.4	0.005	0.4 ± 0.8	0.16
Heart Failure	3 (2.1%)	6.45 ± 2.5	0.08	0.45 ± 0.6	0.7
Chronic Kidney Disease	7 (4.9%)	6.8 ± 1.4	0.001	0.7 ± 0.5	0.8
Obstructive Airway Disease	14 (9.8%)	5.1 ± 1.15	0.7	0.5 ± 0.9	0.83
Active Cancer	3 (2.1%)	5.7 ± 0.4	0.13	-0.06 ± 0.77	0.18
Antihypertensives Before Action					
RAASi	62 (53.4%)	5.3 ± 1.3	0.35	0.53 ± 0.9	0.43
THZ	19 (13.2%)	5.6 ± 1.6	0.08	0.4 ± 0.72	0.3
CCb	38 (26.6%)	5.02 ± 1.1	0.33	0.74 ± 1.06	0.3
β-blockers	46 (32.2%)	5.5 ± 1.1	0.04	0.66 ± 0.9	0.54
Alpha Blockers	3 (2.1%)	5.03 ± 1.3	0.8	0.63 ± 0.13	0.9
Loop Diuretics	3 (2.1%)	6.7 ± 2.25	0.03	0.3 ± 0.6	0.5
Medication List Before Action					
Insulin	19 (13.3%)	5.5 ± 1.5	0.27	0.16 ± 1	0.02
Metformin	50 (35%)	5.4 ± 1.4	0.3	0.55 ± 1	0.6
Sulphonylurea	13 (9.1%)	5.5 ± 1.2	0.3	0.3 ± 0.78	0.2
Dipeptidyl Peptidase-4 Inhibitors	9 (6.3%)	5.9 ± 1.5	0.05	0.42 ± 0.76	0.5
Thiazolidinediones	8 (5.6%)	6.4 ± 0.9	0.006	0.36 ± 0.91	0.46
SGLT2i	13 (9.1%)	5.5 ± 1	0.36	0.13 ± 0.7	0.04
NSAID	8 (5.6%)	5.9 ± 2.3	0.35	0.26 ± 1	0.35
Beta-2 Agonists	9 (6.3%)	5.4 ± 1.2	0.51	0.12 ± 0.8	0.09
Proton Pump Inhibitors	29 (20.3%)	5.4 ± 1.1	0.22	0.3 ± 1.1	0.06
Antidepressants	7 (4.9%)	6.5 ± 1.6	0.005	1 ± 1.4	0.24
Salicylates	10 (7%)	5.6 ± 1.04	0.32	0.65 ± 1.32	0.86
Statins	15 (10.5%)	5.4 ± 1.2	0.5	0.21 ± 0.58	0.02

RAASi was the leading antihypertensive treatment administered before any action, succeeded by beta-blockers. Initial levels were marginally elevated in patients treated with beta-blockers and notably elevated in those receiving loop diuretics (5.5 ± 1.1; p=0.03 and 6.7 ± 2.25; p=0.03, respectively). Continuing the list of medications examination, DPP-4, thiazolidinediones, and antidepressants were correlated with elevated index uric acid levels, whereas insulin, statins, and SGLT2i were significantly connected to a lower Δuric acid (0.16 ± 1; p=0.02, 0.21 ± 0.5; p=0.02, and 0.13 ± 0.7; p=0.04, respectively).

Table 2 illustrates the results of specific interventions across various groups and the entire cohort. There were no significant differences in either index or Δuric acid levels associated with any actions related to RAASi, CCb, and βbl. Similarly, the initiation and escalation of THZ revealed comparable initial and Δuric acid levels. However, the initiation of indapamide did result in a notably higher increase in uric acid. Several minor switches between hydrochlorothiazide and indapamide were observed. In one case, a change from indapamide (1.25 or 1.5mg) to hydrochlorothiazide (25mg) led to a decrease in uric acid level. In six instances, hydrochlorothiazide (12.5mg) was substituted with indapamide (2.5mg), resulting in a mean Δuric acid of 0.8 (± 0.5). A comparison between these cases and those without drug switches showed statistical significance (p=0.05), revealing that, despite the minimal occurrences of such THZ drug switches (n = 7), the differences in effects were significant and warranted attention.

**Table 2:** Comparison of initial and Δuric acid levels based on actions, stratified by treatment status THZ: Thiazide or thiazide-like diuretics, RAASi: Renin-Angiotensin-Aldosterone System Inhibitors; CCb: Calcium Channel Blockers which consist of dihydropyridines and nondihydropyridines, β-blockers: Beta-blockers

		Index Uric Acid	Sig.	Δuric acid	Sig.
In total cohort					
Addition of THZ	124	5.1 ± 1.2	0.08	0.63 ± 0.9	0.3
Escalation of THZ	19	5.6 ± 1.6		0.41 ± 0.16	
Addition or escalation of RAASi	111	5.07 ± 1.17	0.1	0.61 ± 0.83	0.8
RAASi dose was not changed or not added	30	5.6 ± 5.6		0.53 ± 1.06	
Cessation or RAASi dose decrement	2	5.5 ± 0.4		0.7 ± 0.42	
Addition or escalation of β-blockers	8	4.8 ± 0.9	0.7	0.84 ± 0.3	0.8
β-blocker dose was not changed or not added	131	5.2 ± 1.3		0.6 ± 0.5	
Cessation or β-blocker dose decrement	4	5.4 ± 1.26		1 ± 0.34	
Addition or escalation of CCb	23	5.5 ± 1.7	0.3	0.75 ± 0.7	0.4
CCb dose was not changed or not added	102	5.1 ± 1.2		0.54 ± 0.8	
Cessation or CCb dose decrement	18	5.3 ± 0.1		0.76 ± 1.12	
THZ naive group					
Indapamide initiated	49	4.95 ± 1.21	0.22	0.85 ± 0.83	0.026
Hydrochlorothiazide initiated	75	5.22 ± 1.2		0.48 ± 0.9	
Thiazide drug switches among the treated group					
Hydrochlorothiazide to Indapamide	6	5.9 ± 2.35	0.87	0.8 ± 0.51	0.05
Indapamide to Hydrochlorothiazide	1	6.1		-0.8	
Dose escalation without drug change	12	5.52 ± 1.24		0.3 ± 0.7	

Table 3 outlines the relationships between initial laboratory parameters, revealing a mild correlation between initial glucose levels and initial uric acid. Initial creatinine, urea, eGFR, serum K levels, and triglycerides also showed mild, yet significant, correlations with initial uric acid.

**Table 3:** Correlation between initial biochemistry levels and initial and Δuric acid level *Sig: Significance is determined by Spearman's rank correlation test. GFR: Glomerular filtration rate, HbA1c: Glycated hemoglobin, LDL: Low-density lipoprotein

Laboratory parameters	Index Uric acid		Δuric acid	
	Correlation coefficient	Sig.*	Correlation coefficient	Sig.*
Index Uric Acid	1		-0.12	0.17
Delta Uric acid	-0.12	0.17	1	
Index serum glucose corrected Na	-0.1	0.2	0.1	0.2
Index serum K	0.2	0.01	0.09	0.26
Index albumin corrected Ca	0.87	0.46	0.02	0.86
Index creatinine	0.3	<0.001	0.04	0.5
Index estimated GFR	-0.2	0.02	-0.13	0.13
Index urea	-0.28	0.001	-0.16	0.06
Index glucose	0.04	0.6	0.17	0.049
Index HbA1c (present in 99 patients)	-0.08	0.42	0.17	0.09
Index LDL (present in 135 patients)	0.022	0.8	0.028	0.75
Index triglycerides (present in 128 patients)	0.31	<0.001	-0.11	0.23

Machine learning models

**Figure 2:**
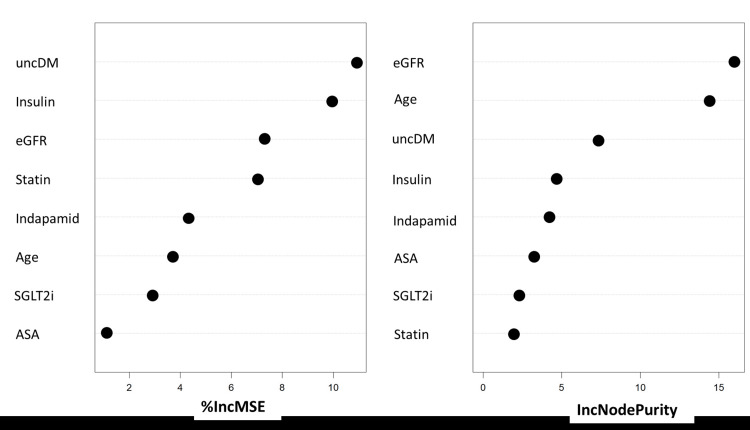
The eight most important features contributing to the Δuric acid slope uncDM: uncontrolled Diabetes, eGFR: estimated Glomerular Filtration Rate, SGLT2i: Sodium-Glucose Cotransporter-2 Inhibitors, ASA: Acetylsalicylic acid, %IncMSE: Percent Increase in Mean Squared Error, IncNodePurity: Increase in Node Purity.

Every conceivable feature was assessed to determine its contribution to a random forest regression model (see the supplementary table in the Appendix). Based on the percentage increase in mean squared error, the top five most crucial features are uncontrolled diabetes, index eGFR level, absence of insulin treatment, initiation or escalation action of indapamide, and absence of statin treatment, among others, as depicted in Figure 2.

Four ML algorithms were employed to ascertain the best fits to the dataset (Table 4). The GBreg model surpassed the others, exhibiting the lowest MAE, MSE, RMSE values, and the highest R2 value (0.779). The least effective performance was noted in the SVM model (R2=0.279). Random forest regression ranked third (R2=0.494), while the NNreg approach was nearly aligned with the GBreg algorithm in evaluating each feature (R2=0.57). This evaluation considered increases in mean squared error and improvements in node purity.

**Table 4:** Performance metrics for each machine learning algorithm

	Mean Squared Error	Root Mean Squared Error	Mean Absolute Error	R^2^
Random Forest Regression	0.39	0.625	0.48	0.494
Gradient Boosting Regression	0.171	0.413	0.28	0.779
Support Vector Machine Regression	0.557	0.746	0.556	0.279
Neural Network Regression	0.326	0.571	0.39	0.57

## Discussion

This study identified co-determinants that could potentially intensify or attenuate the elevation of uric acid when THZ is commenced or the dose escalated in hypertensive patients. To the best of our knowledge, this is the first study to utilize ML algorithms to assess the magnitude of uric acid change associated with THZ and to delineate the contributing factors.

The three most important features affecting THZ-related uric acid rise include having uncontrolled diabetes, lower eGFR, and the existence of insulin at the time of “THZ action”. Having a lower eGFR level, indicative of chronic kidney disease, is logically coherent since uric acid is predominantly excreted through the kidneys. Consequently, uric acid clearance would be correlated with creatinine clearance [17, 18]. Also, individuals with moderately low eGFR would potentially be more susceptible to volume depletion. However, the relationship might not be linear, as those with advanced nephron loss might not respond to thiazides, making volume contraction less of a concern for this group and volume contraction becomes a less pronounced problem than increased volume loads. However, this study does not include advanced chronic kidney disease, and any unresponsiveness is beyond its scope.

Our findings regarding insulin use and diabetes contribute to the ongoing debates surrounding insulin’s multifaceted effects on uric acid levels. At a preliminary glance, insulin seems to elevate renal uric acid reabsorption by enhancing the expression of renal urate transporter 1 (URAT1) and glucose transporter-9 (GLUT9), and by altering the levels of ATP-binding cassette subfamily G member 2 (ABCG2) [19]. Notably, while insulin clamp tests in healthy individuals did not elevate serum uric acid levels, they did result in a significant reduction in urinary uric acid excretion [20]. In this scenario, a marked correlation was observed between diminished fractional uric acid and sodium excretion, inducing a net sodium retention, a recognized outcome of insulin exposure [21]. On the flip side, poor glycemic control and insulin resistance, typical in type 2 diabetes mellitus, are linked with elevated serum uric acid levels and reduced uric acid excretion [22, 23]. A smaller intervention study revealed that both troglitazone treatment and low-energy diets reduced uric acid levels in hypertensive patients [24]. The reciprocal cause-effect relationship also exists as uric acid is known to impede insulin signalling and induce insulin resistance [25]. Accordingly, reducing uric acid levels with febuxostat and allopurinol notably ameliorated insulin resistance [26-29].

In this context, effective insulin utilization and better glycemic control could potentially lower serum uric acid levels by enhancing insulin sensitivity and possibly altering the renal management of uric acid. With precision, it's crucial to note that these discussions pertain to the dynamics of uric acid when either a diabetic or a healthy individual is exposed to exogenous insulin. However, our study explores insulin's influence on the uric acid retaining effect of THZ. Whether it serves as a marker of heightened diabetes control or due to the associated reduction in glucotoxicity with improved insulin sensitivity, insulin seems to alleviate the uric acid retention associated with THZ. Indeed, the expanding corpus of knowledge indicates that THZ can induce hyperuricemia not only through their direct impact on organic anion transporters but also due to the ensuing volume depletion they cause [30-32]. Volume contraction induces H+ ion secretion due to increased sodium sodium-hydrogen exchanger-3 (NHE3) activity, and consequently, and subsequently, the increased cellular pH propels urate uptake through organic anion transporter-4 (OAT4) due to the amplified urate/hydroxyl ion exchange [31]. Corroborating this assertion, it appears that when volume depletion associated with diuretic use is balanced with adequate volume repletion, the occurrence of uric acid retention is averted [33]. Exogenous insulin use, hypothetically, may counteract the volume depletion associated with THZ by promoting tubular sodium reabsorption, hence indirectly reducing the rise in uric acid linked to volume depletion.

Another striking finding involves the ameliorative impact of pre-existing SGLT2i treatment, prior to the “THZ action,” on the elevation of uric acid. Sodium-glucose co-transporter-2 inhibitors (SGLT2i) are relatively new antidiabetic medications that have managed to capture the attention of physicians regularly treating diabetes, as they offer enhanced survival benefits in patients with high cardiovascular risk and heart failure [34, 35]. Moreover, they have earned a reputation as efficacious nephroprotective agents, effectively treating albuminuria [36]. The beneficial profile of SGLT2i extends further, evidenced by meta-analytical data revealing that the administration of SGLT2i induces a uricosuric effect, denoting a reduction in uric acid concentrations, ranging between 0.6 to 0.7 mg/dl [37]. These agents possess mild diuretic and antihypertensive properties; one study exploring the metabolic impacts of transitioning from low-dose THZ to SGLT2i confirmed no elevation in blood pressure while effectively decreasing uric acid levels [38]. The increase in uricosuria and the corresponding decrease in circulating uric acid attributed to SGLT2i are likely due to the inhibition of GLUT9b activity, a transporter located on the apical membrane of renal tubular cells responsible for the transport of both uric acid and d-glucose [39]. SGLT2i also inhibits the activity of URAT1 channels, a primary route for THZ to enter the lumen of the proximal tubule and one of the channels facilitating urate retention [40, 41]. This intersection is accountable for the ameliorative impact of SGLT2i on the uric acid rise related to THZ, a process with many subtleties.

Our research has also illustrated the mitigating impact of statins on the uric acid elevation associated with THZ. A moderate uric acid-reducing effect has been evidenced for atorvastatin and simvastatin, while no such impact was observed for rosuvastatin, and pitavastatin may actually elevate serum uric acid levels [42-44]. Given the diversity within the class of statins, it is unfeasible to conduct post hoc analysis in our cohort to discern which specific statin was associated with the least rise in uric acid. One hypothesis suggests that the diminished serum uric acid levels may be attributed to the augmentation of renal blood flow, stemming from the enhanced endothelial function conferred by atorvastatin [42]. Thus, patients treated with atorvastatin might experience slightly superior renal perfusion and a correspondingly lesser elevation in uric acid levels.

Another subtle yet intriguing observation is that low doses of salicylates, commercially available in 81-100mg, appeared to slightly intensify the magnitude of uric acid elevation in our study. This outcome is anticipated, considering salicylates are recognized to exhibit a bimodal effect on uric acid metabolism [45-47]. High doses of salicylate inhibit URAT1 in a manner akin to losartan and probenecid, leading to uricosuria [48]. Conversely, the utilization of low-dose salicylates, the only form observed in our cohort, inhibits URAT1, resembling the action of pyrazinamide [49]. The heightened URAT1 activity might synergize with THZ-associated uric acid retention, contributing to an elevated concentration of uric acid.

Lastly, our results reveal a more pronounced increase in uric acid levels with indapamide compared to hydrochlorothiazide, and regression analyses have significantly underscored its contribution to the uric acid slope. Current literature provides limited comparisons of uric acid elevations between thiazide and thiazide-like drugs. However, a well-conducted, albeit older, randomized controlled study did illustrate a minor but significant difference [50].

We recognize both the strengths and limitations of our research. A key strength is its distinction as the first study to apply multiple machine learning (ML) regression algorithms for a comprehensive analysis and comparative evaluation of factors affecting THZ-related uric acid changes. While there have been machine-learning studies in nephrology, ours stands out as a pioneer in this specific area, also offering valuable preliminary data that could enhance the rational and effective use of THZ [51]. However, the study's retrospective nature introduces inherent limitations, including selection and recall biases, and the omission of critical variables like BMI, obesity, metabolic-associated fatty liver disease (MAFLD) presence, and gout flare data post-THZ initiation or intensification. Variations in laboratory measurements due to different analytical tools and settings across clinics posed another challenge, although this likely had a minimal impact on uric acid change analysis due to the offsetting nature of the subtraction operation used. Additionally, the availability of insulin and SGLT2i only to diabetic patients, who don't represent our entire study cohort, introduces potential bias. However, we believe this bias doesn't significantly undermine our results, as other antidiabetic treatments don't affect uric acid levels.

Regarding ML algorithm utilization, we did not perform the train-test dataset for an internal validation process, due to the small sample size which presents unique challenges. However, our study's primary objective was to identify codeterminants influencing uric acid rise associated with thiazide therapy, rather than developing a predictive model. Given the limited number of patients, dividing the cohort into separate training and testing sets would have resulted in insufficient data for reliable model training and validation. We acknowledge this as a limitation of our study and have added a section discussing it. Furthermore, we suggest that future research with a larger dataset could employ a traditional split to validate findings more rigorously. We believe that our approach, under the constraints we faced, was the most suitable for the exploratory nature of our analysis.

## Conclusions

In summary, this study has significantly advanced our understanding of the complex determinants affecting uric acid levels in patients treated with thiazide diuretics. Leveraging the analytical power of machine learning, our study has successfully dissected the multifaceted interactions that govern thiazide-induced uric acid rise. The insights gained from our research are expected to have far-reaching implications in the realms of clinical medicine, fostering a more nuanced and individualized approach to hypertension and hyperuricemia management. Furthermore, our findings lay the groundwork for future research in this field, encouraging the exploration of advanced data-driven methodologies to unravel the complexities of drug-disease interactions. Overall, the outcomes of this research are expected to contribute significantly to the advancement of personalized medicine; such as, including more liberal application of thiazides in patients receiving SGLT2i therapy or increased caution in those on low-dose salicylates.

## Appendices

**Table 5:** Ranking Features GFR: Glomerular filtration rate; SGLT2i: Sodium-Glucose Cotransporter-2 Inhibitors; ASA: Acetylsalicylic acid

	Increase in Mean Squared Error	Increase in Node Purity
Age	3.768014	14.209613
GFR	7.326797	15.982299
Uncontrolled DM	10.897961	7.308753
Insulin Treatment	9.908041	4.554556
SGLT2i Treatment	2.788282	2.281675
Indapamide	4.344344	4.121526
Statin	7.167631	1.968991
ASA	1.041819	3.297794
